# Surface Properties and Structural Transformation Behaviors of mPEG-Maleic Rosin Copolymer in Water

**DOI:** 10.3390/polym9100466

**Published:** 2017-09-21

**Authors:** Juying Zhou, Xia Zhang, Yanzhi Zhao, Haitang Xu, Pengfei Li, Hao Li, Jinyan Zhang, Qin Huang, Fuhou Lei

**Affiliations:** 1 School of Chemistry and Chemical Engineering, Guangxi University for Nationalities, Nanning 530006, China; zx1012574109@126.com (X.Z.); zhaoyzsense@163.com (Y.Z.); xhthellen@163.com (H.X.); lipfgxun@126.com (P.L.); lihaospace@163.com (H.L.); zjy_03@126.com (J.Z.); huangqinpeking@163.com (Q.H.); leifuhou@gxun.cn (F.L.); 2 Guangxi Key Laboratory of Chemistry and Engineering of Forest Products, School of Chemistry and Chemical Engineering, Guangxi University for Nationalities, Nanning 530006, China; 3 Key Laboratory of Guangxi Colleges and Universities for Food Safety and Pharmaceutical Analytical Chemistry, School of Chemistry and Chemical Engineering, Guangxi University for Nationalities, Nanning 530006, China

**Keywords:** mPEG-maleic rosin copolymer, surface property, structural transformation, aggregation

## Abstract

mPEG (monomethoxy poly(ethylene glycol))-maleic rosin copolymer was successfully prepared. The surface properties of the copolymer were investigated by surface tension and resonance scattering techniques. The critical micelle concentration (CMC) was obtained. The adsorption behaviors and the conformational changes of the surfactant molecules at the air-water interface were described. The adsorption amount of state 1 presented a sinusoid shape and that of state 2 presented a sigmoid with the growth of *П*. The free energy of adsorption is more negative than that of micellization, thus, the surfactant molecules adsorb on the surface firstly, and then form micelles after saturation adsorption. Accordingly, structural transformation and aggregation behaviors of various concentration mPEG-maleic rosin copolymers with changing temperature were explored in water. The mPEG-maleic rosin chains experienced transformation from unimers to aggregates, to contracted aggregates, to cohesive aggregates with increasing temperature when the concentration is lower than CMC. This process is almost reversible with decreasing temperature. Transformation from micelle to aggregate with increasing temperature happened when the concentration is higher than CMC. The phenomena were assessed by DLS (dynamic light scattering) and SEM (scanning electron microscopy) techniques.

## 1. Introduction

Water-soluble polymers have attracted a great deal of attention over the years for industrial applications, such as dispersants, stabilizers, emulsifiers, and flocculants [1,2]. The representative synthetic examples include poly(ethylene glycol), poly(vinyl alcohol), poly(meth)acrylate, poly(meth)acrylamide, and poly(vinyl ether). Poly(ethylene glycol) (PEG) is a cheap, neutral, water-soluble, biocompatible, FDA-approved polymer and, thus, poly(ethylene glycol) and its derivatives show potential applications in biotechnology and medicine delivery due to their solubility, nontoxicity, low fouling, and biocompatibility. For example, PEG can be used in precipitating proteins [3], excluding proteins and cells from surfaces [4], reducing immunogenicity and antigenicity [5], and preventing degradation by mammalian cells and enzymes [6]. 

Meanwhile, PEG is a compound which has often been utilized as a conjugated segment to other hydrophobic polymer in copolymer synthesis, and its high hydration capacity is favorable for regulation of the hydrophilicity of the materials. PEG-based thermoresponsive polymers, such as copolymer based on poly(vinyl ether), poly(norbornene), polyester, polystyrene, poly(acrylate), or poly(meth)acrylate have been proposed as interesting alternatives to Poly(*N*-isopropylacrylamide) (PNIPAM) and they display reversible phase transitions as a function of temperature or exhibit defined lower critical solution temperature (LCST) in aqueous or physiological media [7]. The phase transitions were caused by the delicate balance between the hydrophilicity and the hydrophobicity of the polymers [8]. Amphiphilic PEG-based copolymers can also form micelles with hydrophilic PEG as a shell and a hydrophobic segment as the core, which were usually applied in biomedical areas. For example, paclitaxel was covalently connected to monomethoxy-poly(ethylene glycol)-*b*-poly(lactide) and released from the conjugate without losing cytotoxicity [9]. Poly(l-glutamic acid) monomethoxy poly(ethylene glycol) (PLGA–mPEG) copolymer nanoparticles were used as nonocapsules in blood circulation [10]. Zhang et al. synthesized a series of triblock copolymers (polyethylene glycol monomethyl ether)-block-poly(e-caprolactone)-block-poly(2-(dimethylamino) ethyl methacrylate), and investigated the self-assembly behavior of these copolymers, which were used in drug loading and release [11]. In addition to the above-mentioned mPEG copolymers, other copolymers, such as mPEG–chitosan diblock copolymer [12], mPEG-PCL (polycaprolactone) diblock copolymer micelles [13], and mPEG-*b*-PCL-grafted chitooligosaccharide (COS-*g*-PCL-*b*-mPEG) copolymers [14], have also been produced and successfully used in drug release.

Rosin is a thermoplastic solid resin that occurs naturally in oleoresins of pine trees [15]. Rosin has fair biodegradation and biocompatibility characteristics, as a class of renewable polymerizable monomer, rosin is not soluble in water, but by introducing hydrophilic moieties the rosin-derived polymers become water soluble. For example, non-ionic surfactants based on rosin-imide maleic anhydride adducts were applied as petroleum crude oil sludge dispersants [16]. Tang’s group synthesized amphiphilic poly(ethylene glycol) and poly(dehydroabietic ethyl methacrylate) block copolymers, which exhibited superior efficacy in impeding tumor growth [17]. Amphiphilic rosin-based copolymer can undergo conformational transition in water at an optimum balance of hydrophilic and hydrophobic moieties. For instance, in our laboratory, amphiphilic dehydroabietic acid-trimethylolpropane ester and acrylicpimaric acid-PEG ester were synthesized by direct covalent conjugation of dehydroabietic acid and acrylicpimaric acid with trimethylolpropane (TMP) and PEG, respectively, and relevant micellization and structural transformation processes were detected in water [18,19].

In this study, mPEG-maleic rosin copolymer was synthesized. The surface properties, including critical micelle concentration, the minimum surface area of molecules, and the thermodynamic parameters of micellization, have been studied. In the meantime, the structural transformation behavior at the molecular level has also been revealed with the RS technique.

## 2. Materials and Methods 

### 2.1. Materials

**Figure 1:**
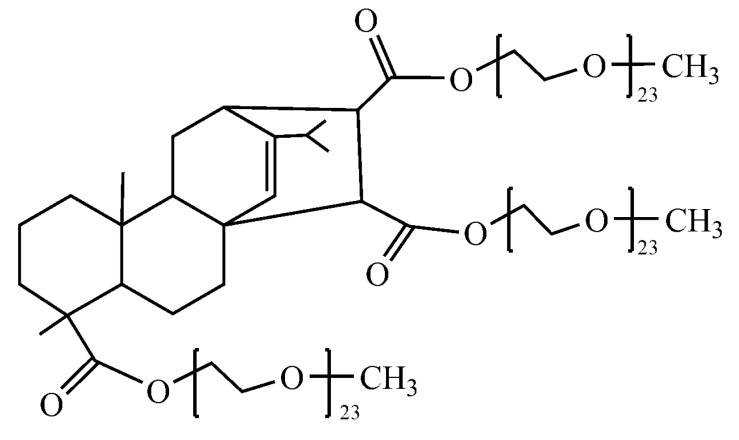
Schematic illustration of mPEG-maleic rosin copolymer.

The mPEG (monomethoxy poly(ethylene glycol))-maleic rosin copolymer was prepared in our laboratory, schematically shown in Figure 1. The mean molecular weight is approximately 3500 and the PDI is 1.32, both were determined by the gel permeation chromatography (GPC) method. Doubly-distilled deionized water was used for all sample preparation and dilution. mPEG-maleic rosin copolymer solution (5 mg·L^−1^) was prepared by adding a known weight of mPEG-maleic rosin copolymer in water. Different concentrations of mPEG-maleic rosin copolymer aqueous solutions were obtained by dilution.

### 2.2. Methods

The surface tensions of various concentration solutions were measured with a tensiometer BZY-2 (Hengping Instrument Company, Shanghai, China). All solutions of different concentrations were prepared and stored in closed bottles for 24 h before measurement. The platinum plate was always cleaned and heated to a red/orange color with a Bunsen burner before use. The aging time was at least 20 min. 

UV–Vis spectra were measured with a UV-1800 UV–Vis spectrophotometer (Shimadzu, Kyoto, Japan).

Rayleigh light scattering measurements were taken on a LS-55 Fluorescence Spectrophotometer (Perkin Elmer, Waltham, MA, USA) with a xenon lamp. Excitation and emission slits with a bandpass of 2.5 nm were used for all measurements. The wavelength interval was 0 nm. Temperature-dependence measurements were carried out with the aid of program-controlled closed-cycle water bath equipment, so that the temperature could be maintained with an accuracy of 0.1 °C. The heating rate was 1 °C·min^−1^. All the spectra were repeatedly recorded three times to ensure perfect duplication.

Dynamic light scattering (DLS) measurements were performed on a Malvern Instruments Zetasizer Nano ZS (Malvern Instruments Ltd., Southborough, Mass, UK) with a detection angle of 90°. The solutions were filtered through a 0.45 μm syringe filter to remove dust particles before measurements. Variable temperature DLS was conducted at the interval of 5 °C. At each temperature the sample was thermally equilibrated for 2 min. The *R*_h_ was obtained by the Malvern software (DTS, Malvern Instruments Ltd., Southborough, MA, USA).

Scanning electron microscopy (SEM) was performed to provide structural details of the mPEG-maleic rosin copolymer using a ZEISS SUPRA 55 (Carl Zeiss, Oberkochen, Germany). A small drop of mPEG-maleic rosin copolymer solution was deposited on copper surface at 40, 60, 80, and 85 °C, respectively, and dried at the corresponding temperature for at least 48 h. The samples were coated with a very thin layer of platinum before measurements were taken.

## 3. Results and Discussion

### 3.1. Equilibrium Surfactant Tension 

**Figure 2:**
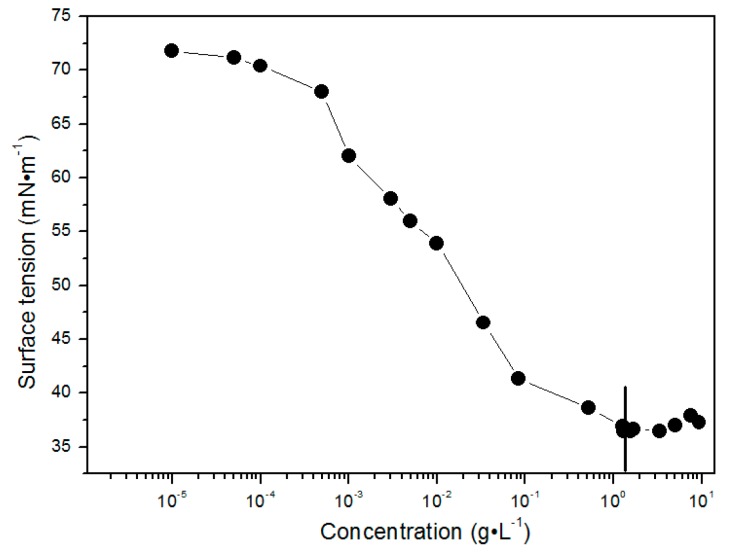
Surface tension changes of mPEG-maleic rosin as a function of concentration.

The CMC value can be obtained from surface tension measurements of the micellar solutions. As shown in Figure 2, the surface tension decreases slowly at lower concentrations, then a steep descent of surface tension is detected at moderate concentrations, and the surface tension levels off at high concentrations. It is worth noting that the interaction point at 1.5 g·L^−1^ of the *γ* vs. *C* plot is estimated to be the CMC of the mPEG-maleic rosin copolymer.

**Figure 3:**
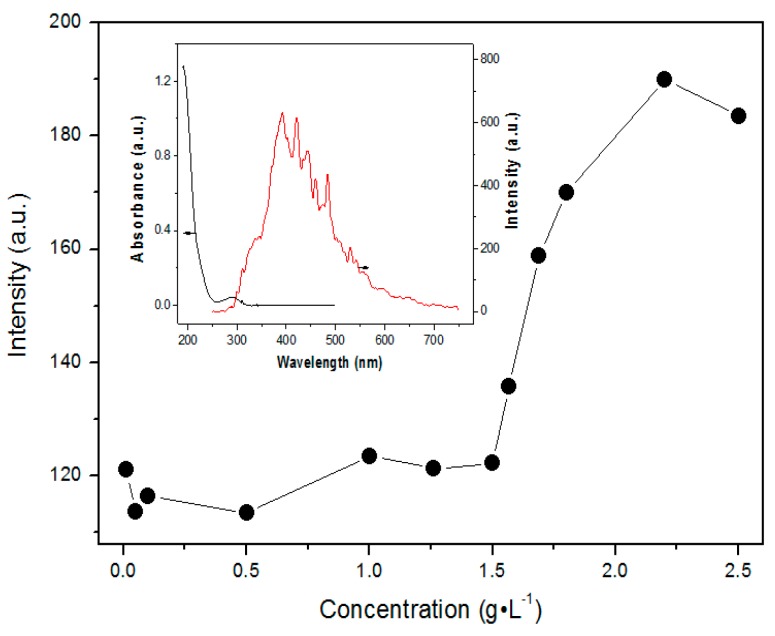
Plot of the Rayleigh scattering intensity at 390 nm versus concentration of the mPEG-maleic rosin. The inset is the Rayleigh light scattering and absorption spectra of the mPEG-maleic rosin copolymer solution.

It can be seen that mPEG-maleic rosin copolymer presents an absorption curve in the UV region, whereas the absorbance approaches almost zero in the range over 300 nm. Thus, the maximum Rayleigh light scattering intensity appears at the red side of the absorption region of the system (about 390 nm). Rayleigh light scattering intensity is widely used for the determination of the CMC of micellar systems. The technique is based on significant changes of scattering intensity at the CMC. Figure 3 displays the RS intensity at 390 nm of mPEG-maleic rosin copolymer solutions with different concentrations, which varied from 0.5 × 10^−4^ to 3 g·L^−1^. At lower concentrations, the intensity retains a roughly constant value, then the intensity increases characteristically with concentration, which is attributed to aggregation of mPEG-maleic rosin copolymer chains. Accordingly, the CMC was determined, which is approximately equal to that obtained from the surface tension technique. It is observed that the CMC value of mPEG-maleic rosin copolymer is much higher than that of monomethyl PEG-550 ester of dehydroabietic acid (0.06 g/L) and monomethyl PEG-750 ester of dehydroabietic acid (0.073 g/L) [20]. This phenomenon is due to more hydrophilic groups and strong hydrophilcity of the mPEG-maleic rosin copolymer. 

### 3.2. Adsorption Behaviors and Conformational Changes of mPEG-Maleic Rosin Copolymer at the Air-Water Interface

Reorientation theory, which was derived from the Butler equation, provides a reasonable description of the behavior of surfactant molecules at the air-water interface. For simplicity, we assume that there are only two different states in the adsorption layer, 1 and 2, respectively. In state 1, the majority of adsorbed surfactant molecules present a state with large partial molar surface area *ω*_1_ at lower concentration. That is, the adsorption layer consists of molecules only in state 1 when surface pressure is 0, i.e., *П* ≈ 0 mN·m^−1^. On the contrary, the increase of concentration leads to a preferential accumulation of states occupying a minimal molar area of *ω*_2_, i.e., state 2. The adsorption layer consists of molecules only in state 2 when *П* > 30 mN·m^−1^.

The adsorption amount in state 1 (*Г*_1_) can be obtained through the surface tension results and Equation (1), which is derived from the Gibbs-Duhem formula [21]. The adsorption amount in state 2, i.e., *Г*_2_ can be gained when the concentration is near CMC:

(1) $$-\frac{d\gamma }{RT}=\Gamma d\;ln\;a$$

where *γ* is the surface tension, *R* and *T* are the gas constant and temperature, respectively, *Γ* is the adsorption amount of surfactant at the air-water interface, and *a* is the activity of the surfactant in the solution. For dilute solutions, *a* can be substituted by the surfactant concentration *c*. Therefore, Equation (1) can be specified by:

(2) $$-\frac{d\gamma }{RT}=\Gamma d\;ln\;c$$

The partial molar surface area occupied by each adsorbed surfactant molecule can be calculated from *Γ* [22]:

(3) $$\omega =\frac{1}{N_{A}\Gamma }$$

*N_A_* is the Avogadro constant. Therefore, *ω*_1_ is 6.68 × 10^6^ m^2^·mol^−1^ and the *ω*_2_ value is 4.36 × 10^5^ m^2^·mol^−1^. These results confirm that the molar area become smaller with increasing surface coverage and stronger competition between the adsorbed surfactant molecules, which reflect the adsorption status of mPEG-maleic rosin copolymer molecules [23]. Accordingly, *A*_1_ and *A*_2_ are equal to 11.1 and 0.725 nm^2^, respectively. 

The molar fractions of solvent *χ*_0_, state 1 *χ*_1_^s^ and state 2 *χ*_2_^s^ change with surfactant concentrations and surface pressures in the surface layer [24].

(4) $$\gamma_{0}-\gamma =-\frac{RT}{\omega_{0}}\ln\chi_{0}{}^{S}=-\frac{RT}{\omega_{0}}(1-\chi_{1}{}^{S}-\chi_{2}{}^{S})$$

(5) $$\ln\chi_{0}{}^{s}=-\frac{(\gamma_{0}-\gamma )\omega_{0}N_{A}}{RT}$$

The surface area of solvent *ω*_0_ can be estimated from the molar area of a bulk-phase H_2_O molecule, e.g., *ω*_0_ = 10^5^ m^2^·mol^−1^ [25]. *χ* is the molar fraction, subscripts 0, 1, and 2 represent the solvent, state 1, and state 2, respectively. The superscript S indicates the surface phase. χ_0_^s^, χ_1_^s^, and χ_2_^s^ satisfy the following relationship:

(6) $$\chi_{0}{}^{s}+\chi_{1}{}^{s}+\chi_{2}{}^{s}=1$$

The molar fractions of solvent *χ*_0_, and the sum of *χ*_1_^s^ and *χ*_2_^s^ can be obtained. 

**Figure 4:**
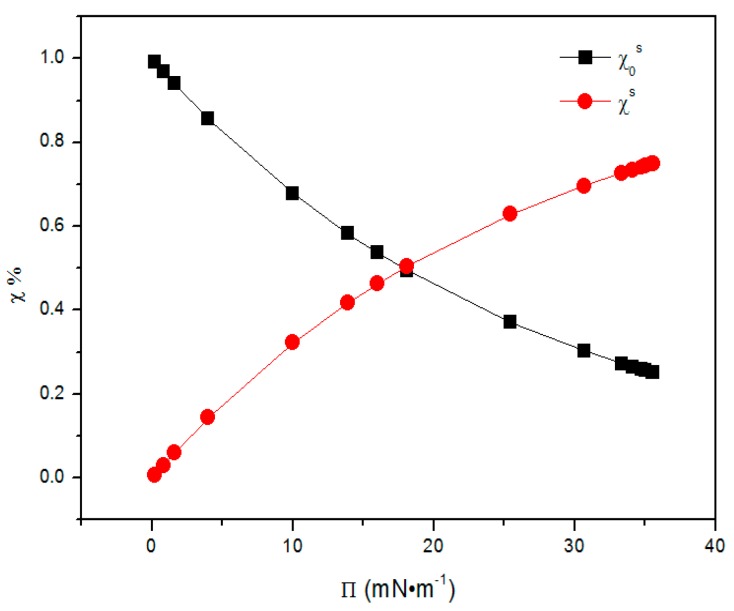
Dependence of the molar fraction of solvent and mPEG-maleic rosin copolymer.

It can be seen from Figure 4 that χ_0_^s^ decreases with increasing *Π*, and the sum of molar fractions of state 1 and 2, i.e., *χ*^s^, increases with increasing *Π* due to the increase of the surfactant molecules at the air-water interface. The transformed surface pressure for mPEG-maleic rosin copolymer is approximately 18 mN·m^−1^. 

The equation involving the ratio of the adsorption amount in the two possible adsorption states can be deduced from the generalized Joos adsorption equation [26]:

(7) $$\frac{\Gamma_{1}}{\Gamma_{2}}=\exp(\frac{\omega_{1}-\omega_{2}}{\omega }){\left(\frac{\omega_{1}}{\omega_{2}}\right)}^{\alpha }\exp[-\frac{\Pi (\omega_{1}-\omega_{2})}{RT}]$$

The total adsorption amount *Γ* and the mean molar area *ω* are defined by the following relations:

(8) $$\Gamma =\Gamma_{1}+\Gamma_{2}$$

(9) $$\omega \Gamma =\omega_{1}\Gamma_{1}+\omega_{2}\Gamma_{2}$$

**Figure 5:**
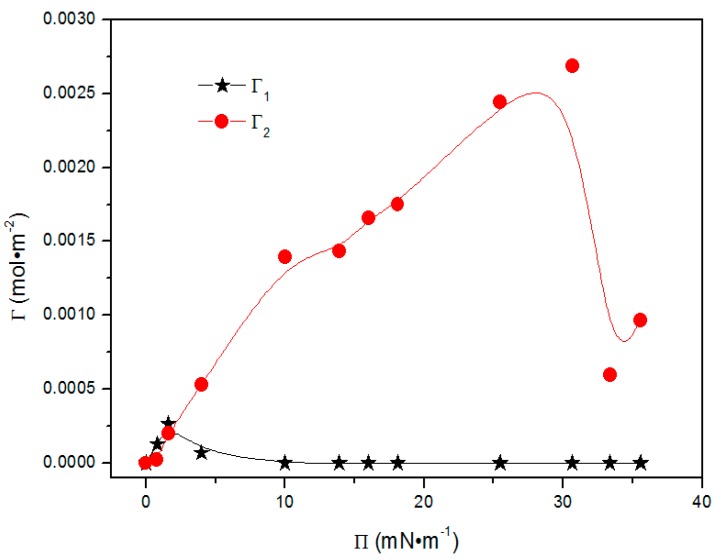
Dependence of the adsorption amount in state 1 and state 2 as a function of surface pressure for mPEG-maleic rosin copolymer.

It was noted that *α* is a constant related to the additional surface activity of state 1. For non-ionic surfactants, *α* = 0 [27]. Thus, the *Π*–*Γ*_1_ and *Π*–*Γ*_2_ curves can be obtained. The dependence of the adsorption amounts in states 1 and 2 on surface pressure are shown in Figure 5.

Clearly, *Γ*_1_ first increases, and then decreases, with increasing *Π*. the *Γ*_1_ curve presents a sinusoid shape, the peak appears at about 1.3 mN·m^−1^. Meanwhile, it is observed that the *Γ*_1_ value becomes almost constant when *Π* exceeds 10 mN·m^−1^. The *Γ*_2_ curve nearly presents a sigmoid shape where the *Γ*_2_ increases until *Π* reaches 30 mN·m^−1^, and *Γ*_2_ decreases when *Π* exceeds 30 mN·m^−1^. It is also discovered that *Γ*_1_ is larger than *Γ*_2_ when *Π* is below 1.3 mN·m^−1^. *Γ*_2_ begins to exceed *Γ*_1_ when *Π* is higher than 1.3 mN·m^−1^. The variations of *Γ*_1_ and *Γ*_2_ reflect the change of the ratio of molecules in states 1 and 2, showing the evolution of molecular states. It is also found that the adsorption amounts, i.e., both *Γ*_1_ and *Γ*_2_, are much higher than that of oxyethylated surfactants studied by Miller’s group [28], which may indicate mPEG-maleic rosin copolymer exhibits better efficiency to reduce surface tension.

### 3.3. Free Energies of Adsorption and Micellization 

Surfactant molecules adsorb on the surface, firstly, and then form micelles after saturation adsorption. Δ*G*_mic_ is the molar standard free energy of micellization of surfactants, which is calculated by the equation valid for nonionic surfactants [26]:

(10) $$\Delta G_{\mathrm{mic}}=RTln\;c_{\mathrm{CMC}}$$

where the *c*_CMC_ is the critical micelle concentration. The molar free energy of adsorption Δ*G*_ad_ is calculated via the following equation:

(11) $$\;\Delta G_{\mathrm{ad}}=\Delta G_{\mathrm{mic}}-0.6023\Pi_{\mathrm{CMC}}A_{\min}$$

where *A*_min_ = *A*_CMC_ = *ω*_2_. Thus, Δ*G*_mic_ of mPEG-maleic rosin copolymer is equal to −24.406 kJ·mol^−1^ indicating that the micellization process is spontaneous. Δ*G*_ad_ is −40.31 kJ·mol^−1^, the value is more negative than Δ*G*_mic_, demonstrating that the adsorption at the surface is associated with a decrease in the free energy of the system. The result may be due to the steric effect of the phenanthrene ring on the inhibition of micellization more than on adsorption. 

### 3.4. Structural Transformation and Aggregation Behaviors of mPEG-Maleic Rosin Copolymer in Water

**Figure 6:**
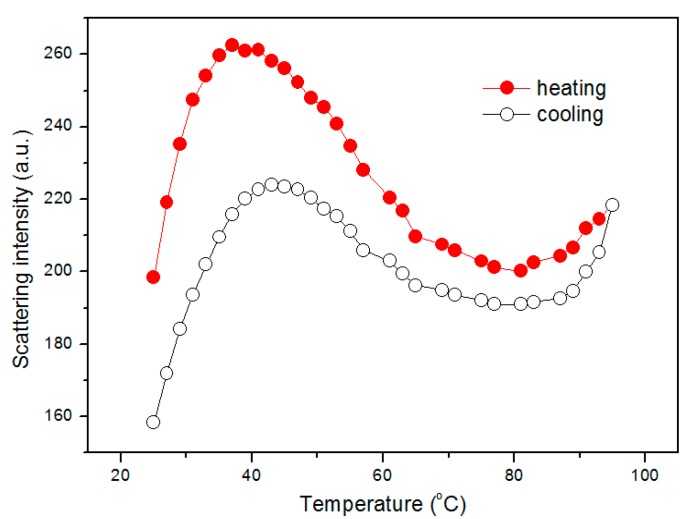
Scattering intensity of 0.13 g·L^−1^ mPEG-maleic rosin solution at 390 nm as a function of temperature during heating and cooling process.

Here, the structure transformation processes of mPEG-maleic rosin copolymer solutions below and above CMC with changing temperature were detected. Figure 6 depicts the temperature dependence of the scattering intensity at 390 nm for 0.13 g·L^−1^ mPEG-maleic rosin copolymer in the temperature range of 25–95 °C. It is worth noting that the inflection points on the curve are associated with different stages of structural transformations upon heating. These stages roughly include structural transformation and aggregation accompanied with the size change of the scatterers. mPEG-maleic rosin copolymer chains exist as individual unimers in water at 25 °C due to the fact that the concentration is lower than CMC. As shown in Figure 6, the first stage is in the temperature region from 25 to 38 °C. In this stage Rayleigh scattering intensity increases rapidly and individual unimers aggregate together with increasing temperature. Here, the aggregations are composed of a hydrophobic core with a maleic rosin segment, carrying hydrophilic mPEG segments in the periphery to ensure solubility. The reason for this phenomenon is that the hydrophobic interactions between maleic rosin moieties increase with increasing temperature. As the temperature is raised further, the scattering intensity decreases in the temperature range from 38–81 °C. The reduction in intensity suggests that the size of the aggregations formed in the previous stage begins to decrease. Owing to relaxation of local mPEG molecular chain segments, the aggregation dimension shrinks and a reduction in the scattering intensity is observed. It is worth noting that in this temperature range the hydrogen bonds between mPEG chains and water begin to break and dehydration takes place with increasing temperature and balance between hydrophilicity and hydrophobicity is once again established, leading to contracted aggregates dispersing in water stably. An enhancement in the scattering intensity is observed again when temperature exceeds 81 °C. This phenomenon is probably attributed to the LCST behavior of mPEG chains in water [29]. Here, further inter-cohesion of contracted aggregates were obtained, which results from the sustained disruption of the hydrogen bonding network between ether oxygen groups and water molecules. The Rayleigh scattering intensity variation for 0.13 g·L^−1^ mPEG-maleic rosin solution was collected during the cooling process, the results are also shown in Figure 6. It can be seen that the intensity presents a decrease at the initial stage of cooling, which is in the temperature range of 95–81 °C. Here, the aggregation size reduced. From 81 °C to 43 °C, the intensity increased slowly, indicating that the scatterers’ size increased. Then the intensity decreases when the temperature is lower than 43 °C. Clearly, in this temperature range, the scattering intensity variation mainly comes from the inverse transformation process from inter-cohesion aggregates, to shrunk aggregates, to aggregates during cooling. It is worth noting that the transformation temperature from aggregates to unimers in the cooling process is 43 °C, which is higher than that in the heating process, thus, the structural transformation behavior does not overlap completely. Here, the irreversible result may be due to the faster stretch of chains and the formation of hydrogen bonds in concentrated solution with decreasing temperature. The reason for the lower intensity scattering intensity after a heating and cooling cycle is that the cooling rate is rapid and the transformation rate cannot keep up with the cooling process [30].

**Figure 7:**
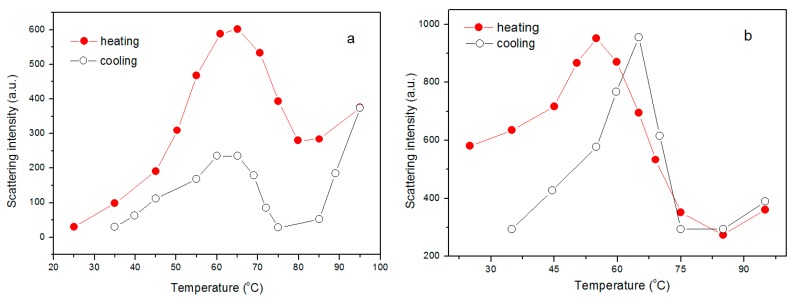
Scattering intensity of 0.6 and 1 g·L^−1^ mPEG-maleic rosin solutions at 390 nm as a function of temperature during one heating and cooling cycle: (**a**) 0.6 g·L^−1^; and (**b**) 1 g·L^−1^.

In order to gain more detailed information about the structural transformation of mPEG-maleic rosin copolymer chains in water, Rayleigh scattering intensity variation for 0.6 and 1 g·L^−1^ mPEG-maleic rosin copolymer solutions were collected during heating, the results are shown in Figure 7a,b, respectively. It is worth noting that similar results were found in these two solutions. It is observed that the transition temperature from aggregation to contracted aggregates is about 65 °C and 55 °C, respectively. The stability of such a copolymer would be a weighted average of these hydrogen bond and hydrophobic contributions. Hydrophobic effects and hydrogen bonding make comparable contributions to the stability of the copolymer at room temperature. It is well known that the formation of the hydrophobic bond is endothermic at low temperature and the strength of the hydrophobic bond increases with increasing temperature. Hydrogen bonds have a temperature dependence opposing hydrophobic bonds. Thus, the irregularity of the transition temperature may come from a different extent of contact between maleic rosin moieties, competition, and predomination of these two kinds of interaction in governing the temperature dependence of stability [31]. However, the transition temperatures from contracted aggregates to inter cohesion of aggregates are almost the same, i.e., 82 °C and 80 °C, respectively, which is almost the same with the results mentioned above. Meanwhile, the Rayleigh scattering intensity variations of 0.6 and 1 g·L^−1^ solutions were collected during the cooling process. A close observation of 0.6 g·L^−1^ mPEG-maleic rosin solution indicates that, in the cooling process, scattering intensity decreases firstly, then the scattering intensity begins to increase at about 75 °C, and finally the scattering intensity decreases again when the temperature is lower than 60 °C. Similar results can be obtained in the 1 g·L^−1^ mPEG-maleic rosin copolymer solution, as shown in Figure 7b. The transformation temperatures from inter-cohesion aggregates to shrunk aggregates almost identify with those in the heating process. The transformation temperature from shrunk aggregates to aggregates for 0.6 g·L^−1^ is equal to that in the heating process. However, the transformation temperature for 1 g·L^−1^ is 64 °C, higher than 55 °C in the heating process. This phenomenon is identified with the results from 0.13 g·L^−1^ mPEG-maleic rosin copolymer solutions. 

**Figure 8:**
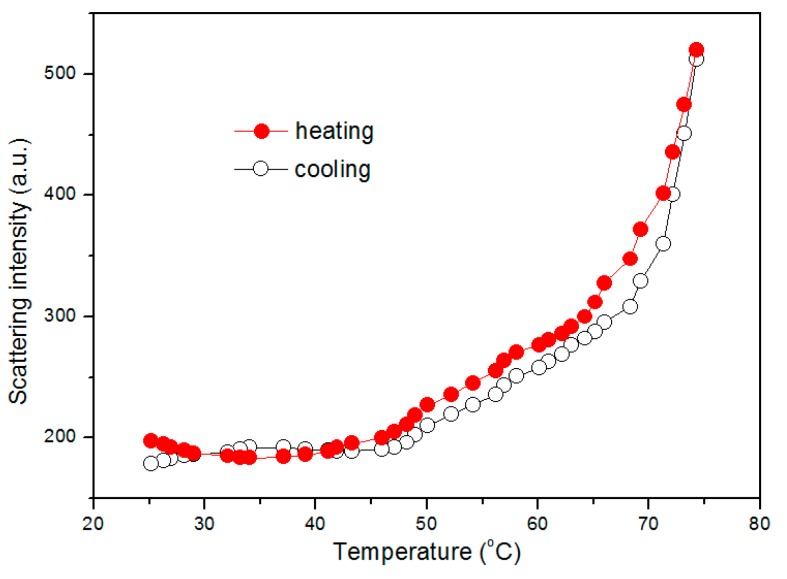
Temperature dependence of I_390_ for 1.62 g·L^−1^ mPEG-maleic rosin solution during one heating and cooling cycle.

Temperature dependence of I_390_ for 1.62 g·L^−1^ of mPEG-maleic rosin copolymer solution (above CMC) during one heating and cooling cycle is plotted in Figure 8. It can be observed that, in the heating process, the scattering intensity remains constant below 45 °C and increases rapidly when the temperature is above 45 °C. In the initial constant period, the micelle is in the equilibrium phase and is stabilized in water, creating no intensity fluctuation due to the moderate repulsive force between hydrophilic groups. The following increase of scattering intensity means further aggregation of micelles, which is assigned to the dehydration of hydrophilic mPEG chains on the peripheral shell. In the following cooling process, the descending of the intensity is almost identical with the heating curve.

**Figure 9:**
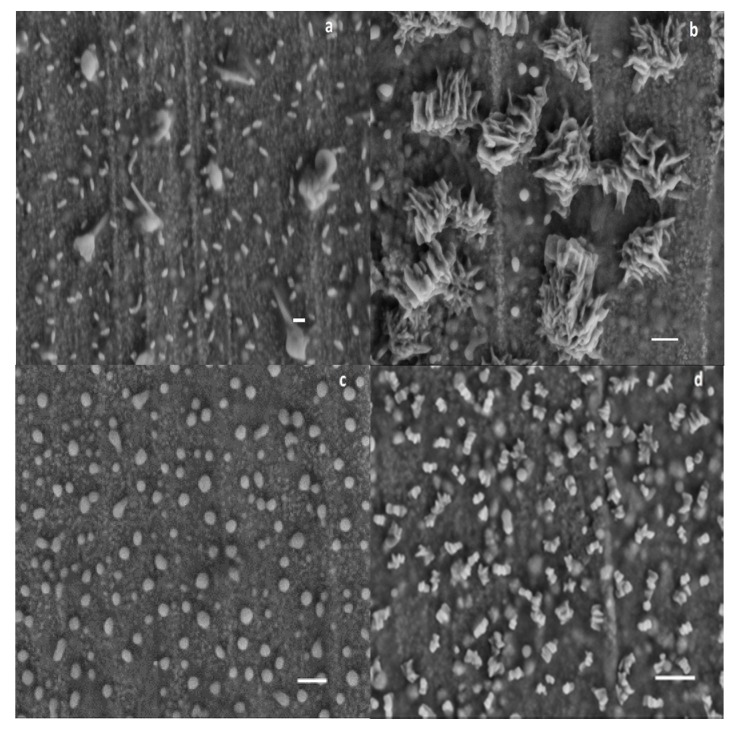
SEM images of the mPEG-maleic rosin aggregates at various temperatures. The deposited temperatures include: (**a**) 40 °C; (**b**) 60 °C; (**c**) 80 °C; and (**d**) 85 °C. Scale bars: (**a**) 100 nm; (**b**,**c**) 200 nm; and (**d**) 300 nm.

To obtain direct evidence of structural transformation and aggregation behaviors, the typical microscopic morphologies of 0.6 g·L^−1^ mPEG-maleic rosin aqueous solution deposited at 40, 60, 80, and 85 °C are provided in Figure 9. It was found that at 40 °C, worm-shaped individual unimers are presented in Figure 9a. A close observation exhibit that there are also some aggregates in this temperature which may indicates that unimers aggregate together in drying. As the temperature increases to 60 °C, the unimers form large aggregates with an average diameter in the range of 200–250 nm (Figure 9b). Spherical aggregates with a diameter of about 50–80 nm appear at 80 °C (Figure 9c), the results mean that the aggregates formed at 60 °C are deformed by heating. Gear-shaped aggregates with a diameter of about 100 nm appear at 85 °C (Figure 9d), which may due to the gathering of spherical aggregates.

**Figure 10:**
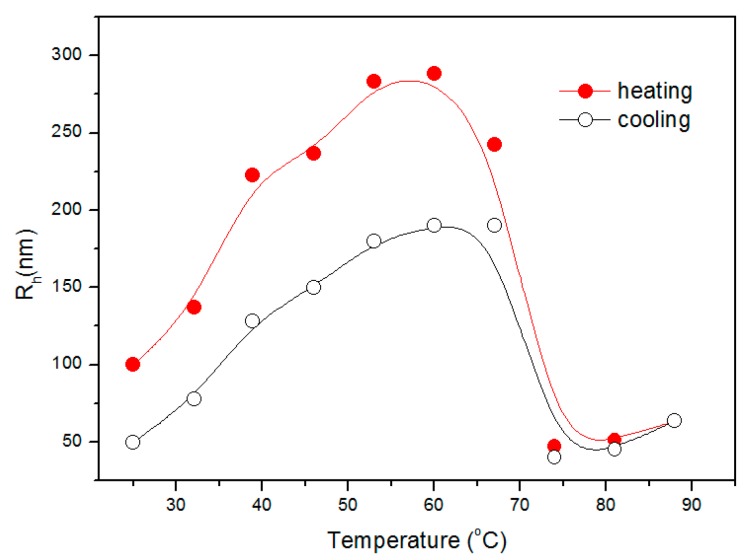
Temperature dependence of *R*_h_ for 0.6 g·L^−1^ mPEG-maleic rosin solution collected during one heating and cooling cycle.

In the meantime, the DLS measurements were performed to study the size variation as a structure transformation. Figure 10 displays the temperature dependence of *R*_h_ of 0.6 g·L^−1^ mPEG-maleic rosin in water. It can be seen that the *R*_h_ is approximately 100 nm at 25 °C. The *R*_h_ then gradually increases when the temperature increases, indicating the aggregation of unimers and then *R*_h_ reaches a maximum of about 270 nm at about 60 °C, it decreases to 50 nm at 75 °C, and then it increases again above 80 °C. In the cooling process *R*_h_ gradually decreases with temperature and then *R*_h_ begins to increase, reaching a maximum at about 67 °C, and decreases in the temperature range of 67–25 °C. These results demonstrate that the *R*_h_ obtained by the DLS method is almost consistent with the results observed by Rayleigh scattering and SEM techniques. The difference of *R*_h_ from DLS and SEM comes from the different testing methods.

**Figure 11:**
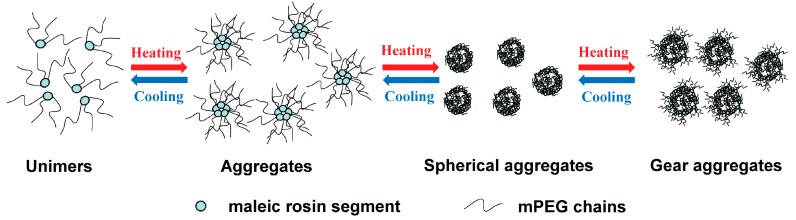
Evolution of structural transformation and aggregation of mPEG-maleic rosin chains during heating and cooling processes.

According to the above analysis, the structural transformation and aggregation of mPEG-maleic rosin chains during one heating and cooling cycle can be simply described as follows: an almost reversible aggregation of unimers occurred at low temperature, and the aggregation process is followed by another reversible transformation process from the larger aggregates to spherical aggregates and to gear aggregates with increasing temperature. A model is proposed to depict the entire structural transformation and aggregation of mPEG-maleic rosin chains during the heating and cooling processes in Figure 11. The morphologies are similar to those of “crew-cut” micelle-like aggregates of polystyrene-*b*-poly(acrylic acid) copolymers in aqueous solutions [32,33]. The thermo-induced multi morphologies transformation of mPEG-maleic rosin chains make it potentially useful as a carrier due to similar characteristics with block copolypeptoids in water [34,35].

## 4. Conclusions

In conclusion, the CMC value of mPEG-maleic rosin was decided by surface tension and scattering techniques. Reorientation theory was proposed to describe the adsorption states, adsorption behaviors, and adsorption processes of mPEG-maleic rosin at the air-water interface. The mPEG-maleic rosin adsorbed on the air-water interface in two different states; they are state 1 and state 2. The molar fraction of solvent decreased, and the sum of molar fractions of these two states increased, with the increasing surface pressure. The adsorption amounts of state 1 presented a sinusoid shape and those of state 2 presented a sigmoid shape with the growth of *П*. Free energies of adsorption are more negative than micellization, which demonstrated the surfactant molecules adsorbing on the surface, firstly, and then forming micelles after saturation adsorption. The structural transformations of mPEG-maleic rosin chains were investigated by RS, SEM, and DLS techniques. Structural transformation from unimers, to aggregates, to spherical aggregates, to gear aggregates with increasing temperature was monitored when the concentration is lower than CMC, which is almost a reversible process during cooling process. Transformation from micelle to aggregate with increasing temperature happened when the concentration was higher than CMC.
